# Household water insecurity, missed schooling, and the mediating role of caregiver depression in rural Uganda

**DOI:** 10.1017/gmh.2017.14

**Published:** 2017-08-15

**Authors:** C. E. Cooper-Vince, B. Kakuhikire, D. Vorechovska, A. Q. McDonough, J. Perkins, A. S. Venkataramani, R. C. Mushavi, C. Baguma, S. Ashaba, D. R. Bangsberg, A. C. Tsai

**Affiliations:** 1 Massachusetts General Hospital, Boston, MA, USA; 2 Harvard Medical School, Boston, MA, USA; 3 Mbarara University Science and Technology, Mbarara, Uganda; 4 Bloomberg School of Public Health, Johns Hopkins University, Baltimore, Maryland, USA; 5 School of Public Health, Oregon Health Sciences University-Portland State University, Portland, Oregon, USA

**Keywords:** Child, depression, schooling, water insecurity

## Abstract

**Background:**

School attendance rates in sub-Saharan Africa are among the lowest worldwide, placing children at heightened risk for poor educational and economic outcomes. One understudied risk factor for missed schooling is household water insecurity, which is linked to depression among women and may increase children's water-fetching burden at the expense of educational activities, particularly among children of depressed caregivers. In this study conducted in rural Uganda, we assessed the association between household water insecurity and child school participation and the mediating pathways behind these associations.

**Method:**

We conducted a population-based, cross-sectional study of female household heads (*N* = 257) and their children ages 5–17 (*N* = 551) in the rural regions surrounding the town of Mbarara, in southwestern Uganda. We used multivariable linear regressions to estimate the association between water insecurity and missed schooling. We then assessed the extent to which the association was mediated by caregiver depression.

**Results:**

Among children, water insecurity had a statistically significant association with the number of missed school days (a standard deviation increase in water insecurity resulted in 0.30 more missed school days in the last week). The estimated association was partially mediated by caregiver depression. When stratified by sex, this mediating pathway remained significant for boys, but not among girls.

**Conclusions:**

Water insecurity is a risk factor for missed schooling among children in rural Uganda. Caregiver depression partially mediated this relationship. Also addressing caregiver mental health in water insecure families may more fully address the needs of sub-Saharan African families and promote educational participation among youth.

Fifty-nine million primary school-aged children worldwide are out of school (UNESCO, 2015), with the lowest rates of attendance in sub-Saharan Africa (UNICEF, 2015*a*, *b*). School attendance is important for healthy child development and is linked to increased earnings and health of offspring later in life (Psacharopoulos & Alam, 1991; Hargreaves *et al.* 2008; Henley *et al.* 2010; Wood *et al.* 2012; Cortina *et al.* 2013; Abdullah *et al.* 2015; Devkota & Panda, 2016; Kanté *et al.* 2016; Mehta & Preston, 2016; Richter *et al.* 2017). In sub-Saharan Africa, missed schooling is associated with child engagement in water fetching (Burke & Beegle, 2004; Hemson, 2007; Dreibelbis *et al.* 2013). This task that is often completed by children, particularly girls, in support of women (Sorenson *et al.* 2011; Graham *et al.* 2016). In one study conducted in rural Ethiopia, nearly 20% of households reported that girls missed school to help with water fetching, while only 5% of households reported that boys missed school for this reason (Stevenson *et al.* 2012). These findings suggest that girls may be at elevated risk for missed schooling in East Africa. As one-third of families in sub-Saharan Africa dedicate over an hour a day to water fetching (Sorenson *et al.* 2011; Graham *et al.* 2016), insecure water access poses a significant risk to child educational outcomes.

Little is known about how household water access drives problems with child schooling attendance. In sub-Saharan Africa, nearly 350 million people lack reliable access to safe drinking water (WHO & UNICEF, 2014). The limited or uncertain availability of safe water acquired via culturally acceptable means, termed water insecurity, is linked to depression and anxiety among women in low- and middle-income countries (LMICs), as they bear the primary water fetching burden globally and in sub-Saharan Africa (Wutich & Ragsdale, 2008; Hadley & Wutich, 2009; Sorenson *et al.* 2011; Stevenson *et al.* 2012; Graham *et al.* 2016; Tsai *et al.* 2016). This distress is in part associated with women's failure to fulfill domestic roles (e.g. washing, cleaning, and cooking), acts of hospitality, and hygiene due to limited water (Stevenson *et al.* 2012), which mirror and may further exacerbate any observed domestic impairments among depressed women (Bolton *et al.* 2002).

In high-income countries, maternal depression is linked to poor child school attendance and academic performance (Guevara *et al.* 2013; Claessens *et al.* 2015). In both high- and low-income countries, poor maternal mental health is a well-established risk factor for adverse child developmental outcomes, which are central to school-readiness and academic performance (Petterson & Albers, 2001; Parke *et al.* 2004; Tsai & Tomlinson, 2012; Choe *et al.* 2014; Verkuijl *et al.* 2014; Ng *et al.* 2015; Tomlinson & Morgan, 2015; Bennett *et al.* 2016; Familiar *et al.* 2016; Huang *et al.* 2017). Further, the cognitive and psychomotor symptoms of depression (i.e. fatigue, psychomotor slowing, impaired concentration/decision-making) in combination with the domestic and childrearing impairments (e.g. cooking meals, bathing children, and laundering clothes) found among depressed mothers in sub-Saharan Africa are likely to undermine their children's ability to arrive at school on time appropriately fed, bathed, and dressed (Cooper *et al.* 1999; Bolton *et al.* 2002) and inhibit parental involvement which is known to support child academic engagement and success (Wang & Sheikh-Khalil, 2014).

No studies have attempted to integrate these disparate strands of literature and assess the extent to which caregiver depression mediates the association between household water insecurity and adverse child schooling outcomes. To address this gap in the literature, we analyzed data from a cross-sectional, population-based sample of female household heads and school-age children in a rural community in southwestern Uganda. We hypothesized that household water insecurity would be associated with child school attendance and this relationship would be mediated by caregiver depression. We also hypothesized that the mediating effect would be stronger for girls.

## Method

### Participants

The present study was conducted in Nyakabare Parish, Mbarara District, Uganda, which is located approximately 260 km southwest of the capital city of Kampala. The Parish is located approximately 20 km outside of Mbarara Town, the district's primary commercial hub. The economy is based primarily on subsistence agriculture, and both food and water insecurity are common (Tsai *et al*. 2011, 2016).

Among the 758 households in the Parish, we identified all 358 households in which there was a child younger than 5 years of age and a woman of reproductive age (18–49 years, or emancipated minors aged 16–18 years) who considered Nyakabare her primary place of residence, and who was available and capable of providing consent for interviewing. In households with multiple eligible women, we interviewed the oldest woman in that age range. Therefore, all women included in this study were female caregivers of the children assessed, though not all were the biological mother of the child. Potential participants meeting these eligibility criteria were approached by a research assistant fluent in the local language (Runyankole) to request their participation in the study. Written informed consent for study participation was obtained from those expressing interest. Those who were not able to provide written consent due to literacy concerns were permitted to indicate consent with a thumbprint. Once enrolled, each study participant completed an individual interview in a private location beyond the earshot of others. Interviews were completed with 257 women who provided data on 551 school-age children (5–17 years) living in their households.

Ethical approval for all study procedures was obtained from the Partners Human Research Committee, Massachusetts General Hospital; and the Institutional Review Committee, Mbarara University of Science and Technology. Consistent with national guidelines, we received clearance for the study from the Uganda National Council for Science and Technology and from the Research Secretariat in the Office of the President.

### Measures

*Water Insecurity* (WI) was measured with the Household Water Insecurity Access Scale (HWIAS; Tsai *et al.* 2016). The HWIAS is an eight-item self-report measure of household water insecurity, with possible total scores ranging from 0 to 24. The items of the HWIAS were developed based on the items of the Household Food Insecurity Access Scale (Swindale & Bilinsky, 2006), and assess perceptions of insufficient quantity or quality of water, feelings of uncertainty or anxiety over water access, and strategies for coping with insufficient water for completing water-based tasks. The HWIAS has been validated for use among Runyankore-speaking populations in Uganda (Tsai *et al.* 2016).

*Caregiver Depression* was assessed with the Hopkins Symptom Checklist-Depression Subscale (HSCL-D). The HSCL-D is a 15-item self-report of depressive symptoms taken from the short form of the HSCL-25, which assesses both anxiety and depression (Derogatis *et al.* 1974). HSCL-D assess the frequency of symptoms of depression in the past 7 days, such as ‘felt weak and low in energy, been blaming yourself when things go wrong.’ The version of the HSCL-D used in our study was modified for the local context; one item from the original scale (‘feeling trapped’) was dropped because it did not perform well in this context, and one item was added (‘don't care what happens to your health’; Bolton & Ndogoni, 2001). This version of the HSCL-D has shown strong evidence of reliability and validity in the local context (Tsai *et al.* 2012; Psaros *et al.* 2015).

*Missed schooling* was assessed for children aged 5–17 years via caregiver report. For children enrolled in school, caregivers were asked ‘In the past 7 days, how many school days did this child miss for any reason?’ and ‘In just the past 7 days, how many days was this child late to school because of fetching water?’

In addition to these measures, the survey asked female household heads about basic demographic characteristics including their age, marital status, level of education, number of children under 18 living in the home, child age and sex, and family assets in the form of consumable goods (e.g. owns a working radio, has a house with walls made of cement). The asset data were used to calculate an index of household asset wealth (Filmer & Pritchett, 1999); absolute values of the asset index cannot be interpreted directly but can be used to rank households by overall wealth in relative terms, with higher values indicating higher levels of asset wealth.

### Statistical analysis

**Fig. 1.**
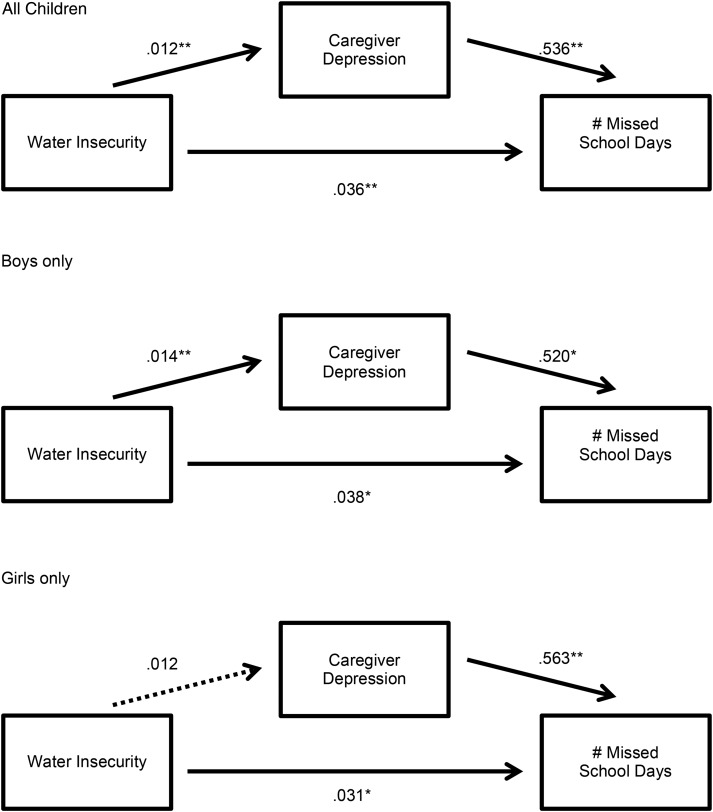
Unstandardized models of caregiver depression as a mediator between water insecurity and missed schooling. Note: *p < 0.05, **p < 0.01

**Fig. 2.**
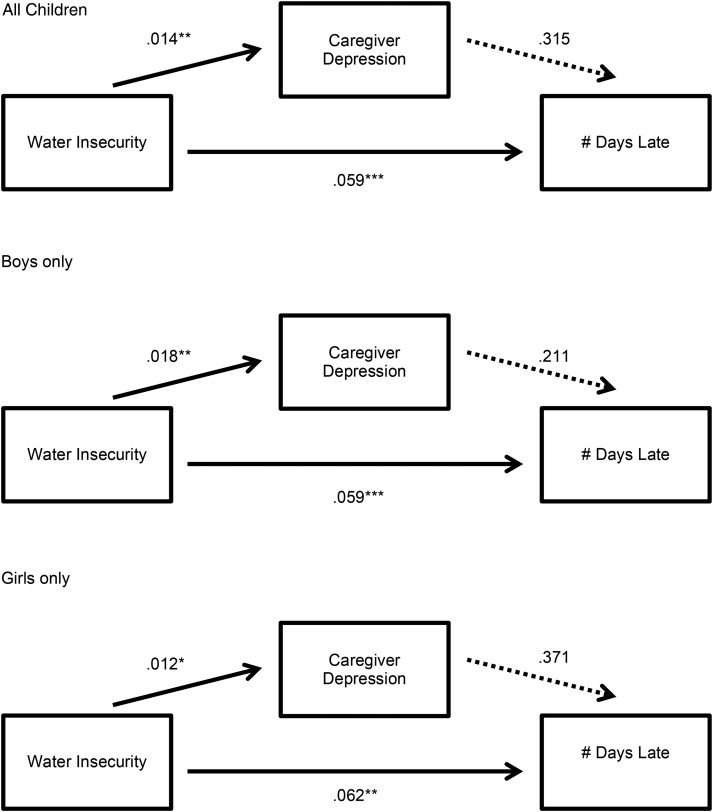
Unstandardized models of caregiver depression as a mediator between water insecurity and school tardiness. Note: *p < 0.05, **p < 0.01, ***p < 0.001.

All statistical analyses were conducted using the Stata/SE software package (version 14.0; StataCorp, 2015). We followed the procedures described by Imai *et al*. (2010*a*, *b*) to estimate the association between household water insecurity and missed schooling, and the extent to which caregiver depression mediated this association. The parametric algorithm described in Imai *et al*. (2010*a*) computes two quantities of interest: the average causal mediation effect, which is the average change in missed schooling corresponding to a change in caregiver depression under less *v.* more exposure to household water insecurity; and the average direct effect, which is the average of all other causal mechanisms linking household water insecurity to missed schooling. As the exposure, outcome, and mediator were all continuous variables, we used standard linear regression. Separate models were used to evaluate the outcomes of number of school days missed (see Fig. 1) and number of days late to school (see Fig. 2) in all children, and then in girls and boys separately. In all analyses, standard errors were corrected for clustering at the household level; and we adjusted for caregiver age, education, marital status, household asset wealth, and number of children in the household.

## Results

### Sample characteristics

Participating women were on average 33.5 years old (s.d. = 7.9). Each woman, on average, lived in a household with three children under the age of 18 (range, 1–8). The mean WI score was 9.9 (s.d. = 6.9). The mean depression score among female household heads was 1.76 (s.d. = 0.49), with 44% scoring above the 1.75 point cut-off used to denote probable depression (Winokur *et al.* 1984). Most women were married or cohabiting with a partner (82%) and had not completed primary education (52%). Among the children described by the caregivers, 49% were girls, and the mean age across all children was 9.2 years (s.d. 3.4). Overall, 42% of children missed one or more school days in the last week and 20% were late to school one or more days in the last week. There were no statistically significant differences in number of school days missed (*t* = 0.54, *p* = 0.59) or number of school days tardy (*t* = −0.83, *p* = 0.40) between boys and girls.

### Number of missed school days

Among all children, there was a statistically significant association between household water insecurity and the number of missed school days (*b* = 0.04; 95% CI 0.02–0.06). Evaluated at the mean values of the covariates, a one standard deviation (s.d.) increase in household water insecurity was associated with a 0.30 increase in the number of missed school days in the last week (0.30/1.31 = 0.23 s.d. increase). Household water insecurity also had a statistically significant association with depression symptom severity among female household heads, which partially mediated the association between water insecurity and missed school days: 16% of that association was mediated by caregiver depression (see Table 1 and Fig. 1).

**Table 1.** Mean effects for caregiver depression mediating the relationship between water insecurity and missed schooling

	Average causal mediated effect (95% CI)	Direct effect (95% CI)	Total effect (95% CI)	% of total effect mediated (95% CI)
Missed days of school				
All children	0.006 (0.001–0.013)	0.036 (0.014–0.057)	0.042 (0.020–0.065)	0.155 (0.099–0.318)
Boys only	0.007 (0.001–0.016)	0.038 (0.007–0.067)	0.045 (0.014–0.077)	0.168 (0.095–0.522)
Girls only	0.007 (−0.0001 to 0.016)	0.031 (0.004–0.056)	0.037 (0.010–0.064)	0.182 (0.102–0.608)
Days late to school				
All children	0.004 (−0.002 to 0.013)	0.059 (0.033–0.083)	0.063 (0.039–0.088)	0.070 (0.050–0.112)
Boys only	0.004 (−0.006 to 0.015)	0.059 (0.030–0.086)	0.062 (0.037–0.090)	0.059 (0.041–0.100)
Girls only	0.004 (−0.003 to 0.015)	0.062 (0.026–0.097)	0.067 (0.032–0.103)	0.068 (0.043–0.137)

When analyses were stratified by child sex, among boys, there was a statistically significant association between household water insecurity and the number of missed school days (*b* = 0.05; 95% CI 0.01–0.08). Evaluated at the mean values of the covariates, a 1 s.d. increase in household water insecurity was associated with a 0.31-day increase in the number of missed school days in the last week (0.23 s.d. units when compared with the sample mean). Household water insecurity also had a statistically significant association with depression symptom severity among female household heads, which partially mediated the association between water insecurity and missed school days (see Fig. 1); 17% of that association was mediated by caregiver depression (see Table 1).

Among girls, there was also a statistically significant association between household water insecurity and number of missed school days (*b* = 0.04; 95% CI 0.01–0.06). Evaluated at the mean of the covariates, a 1 s.d. increase in household water insecurity translated into a slightly smaller effect size of 0.26/1.26 = 0.21 s.d. units. However, the average causal mediated effect was not statistically significant (see Table 1 and Fig. 1).

### School tardiness

Household water insecurity had a statistically significant association with days of school late in the entire sample (*b* = 0.06; 95% CI 0.04–0.09) and translated into slightly larger effect size of 0.44/1.42 = 0.31 s.d. units. When stratified by sex, this finding was significant for both boys (*b* = 0.06; 95% CI 0.04–0.09; 0.32 s.d. units) and girls (*b* = 0.07; 95% CI 0.03–0.10, 0.31 s.d. units). However, because there was no statistically significant association between number of days late and depression among the female household heads, no statistically significant average causal mediation effects were observed (see Table 1 and Fig. 2).

## Discussion

In this cross-sectional, population-based study of female household heads in rural Uganda and their children, we found that household water insecurity had a statistically significant association with missed schooling for children. The estimated association was large and statistically significant, and the effect was partially mediated by caregiver depression. These findings are consistent with the literature linking water insecurity to increased depression among women in LMICs (Wutich & Ragsdale, 2008; Stevenson *et al.* 2012; Tsai *et al.* 2016). Building on a previous work from high-income countries, our study establishes an intergenerational spillover effect leading to poor school attendance in a rural Ugandan community (Guevara *et al.* 2013; Claessens *et al.* 2015). Our analyses adjusted for household asset wealth and other socioeconomic characteristics, so the effects of water insecurity and depression on missed schooling are unlikely to be better explained by lack of financial means for paying school fees. The mediating role of depression among female household heads may potentially be explained by the domestic role impairments associated with water insecurity and depression in eastern Africa (e.g. preparing family meals, washing clothing, and bathing children; Bolton *et al.* 2002; Stevenson *et al.* 2012). Such impairments may increase the chore burden, including water fetching, placed on children, and time spent completing these tasks is likely to delay departure for school and require costly tradeoffs with school attendance. The present study does not allow for examining differences between biological and non-biological caregivers. Findings from qualitative work underway at this site suggest did not identify differences in water procurement demands for biologically and non-biologically related caregivers, though themes suggest that non-biologically related children may disproportionally bear the burden of chores including water fetching.

Notably, when analyses were stratified by sex, we only found evidence of mediation for boys but not girls. This discrepancy could potentially be explained by the increased levels of instrumental support in water fetching that girls tend to provide their mothers, relative to boys (Sorenson *et al.* 2011; Graham *et al.* 2016). This distribution of labor between boys and girls was described by some mothers interviewed in our qualitative investigation at this site, which is consistent with local cultural norms of girls being assigned household chores. However, other mothers described a more equal division of water fetching among young boys and girls. Such support may buffer women against the emotional stress of experiencing water insecurity but at the cost of lowering school attendance for girls. It is possible that the gender specific finding is driven by statistical power. Depression among the female household heads was significantly associated with water insecurity in the mediation models evaluating the outcome of school tardiness, consistent with the previous work (Burke & Beegle, 2004; Hemson, 2007; Dreibelbis *et al.* 2013), but the magnitude and statistical significance were smaller in the model for girls. However, there were an approximately equivalent number of girls and boys, so a difference in statistical power is unlikely to be a sufficient explanation for the divergent findings.

Though we hypothesized that caregiver depression would delay departure for school among their children, we did not find evidence of this phenomenon. It is possible that caregiver depressive role impairments cause more significant disruption to the family routine, resulting in outcome differences along the extensive margin (i.e. children missing an entire day of school) rather than along the intensive margin (i.e. children not missing school but arriving late). Further, unpublished data from our ongoing qualitative study of water insecurity in this setting suggest that Ugandan children who are tardy for school often face harsh punishment and shaming at school, and therefore may prefer to miss an entire day rather than arrive late and face these undesirable consequences. Such treatment at school is consistent with social norms supporting the use of corporal punishment and low mental health literacy found among educators in other parts of sub-Saharan Africa (Lansford *et al.* 2010; Kutcher *et al.* 2015).

The findings of this investigation must be considered in light of several limitations. First, the cross-sectional study design does not allow for the evaluation of temporal precedence in the mediational models. Therefore, these findings should be regarded as preliminary support for the mediating role of caregiver depression, and further evaluation of these models using a longitudinal study design is warranted. Second, the data were all based on self-report and therefore carry an inherent reporting bias. However, it should be noted that in the local setting, because child school attendance is a source of pride for families, at times families may actually under report their child's missed schooling; thus, we expect that our findings potentially underestimate the magnitudes of the effects observed. Third, our measure of depression was based on a symptom checklist, and these are known to overestimate the prevalence of major depressive disorder due to false positives (Kagee *et al.* 2013; Tsai, 2014). Given the study design, we were not able to determine which women were experiencing a major depressive episode. Fourth, the study design did not allow for evaluating differences between biological and non-biological caregivers in the relationships examined. Lastly, we were not able to account for the potential mediating effects of intergenerational transmission of depression from caregiver to child (Bowlby, 1988; Manczak *et al.* 2017; Weissman *et al.* 2016) or of physical illness associated with water insecurity (Fewtrell *et al.* 2005; Hunter *et al.* 2009), which may have also influenced child school attendance.

Taken together, these findings advance our understanding of the processes through which water insecurity undercuts children's school participation in LMICs, with attendant implications for interventions to enhance children's developmental outcomes. Foremost, these findings suggest that structural interventions alone are unlikely to fully alleviate the deleterious effects of water insecurity on child schooling and should consider also seeking to address the mental health needs of caregivers in addition to improving reliable access to clean water. For example, supported parenting and counseling interventions (e.g. Interpersonal Therapy and Cognitive Behavioral Therapy; Bolton *et al.* 2003; Nakimuli-Mpungu *et al.* 2014) delivered alongside structural water interventions could more fully address depressive symptoms and functional impairments associated with water insecurity, and help women engage in problem solving and more adaptive parenting behaviors (Stein *et al.* 2014). In turn, they may be better able to manage domestic role demands, including their family's water collection. Such integrated interventions have the promise to support children's school attendance and achievement to reduce the intergenerational transmission of adverse water insecurity-related outcomes.
